# Attitudes of Surgical Nurses Toward Non-Pharmacological Postoperative Pain Management: A Descriptive Cross-Sectional Study

**DOI:** 10.3390/nursrep16070252

**Published:** 2026-07-20

**Authors:** Çağla Toprak, Eva Kajti, Zahra Ebadi

**Affiliations:** Faculty of Health Sciences, Atlas University, Istanbul 34408, Türkiye; eva.kajti@atlas.edu.tr (E.K.);

**Keywords:** postoperative pain, nonpharmacological methods, surgery, surgical nursing

## Abstract

**Background:** Postoperative pain is a significant continuous clinical problem for patients in surgical units. This study aimed to examine surgical nurses’ attitudes toward the use of non-pharmacological techniques in postoperative pain management and to identify factors associated with these attitudes. **Methods:** This descriptive and cross-sectional study was conducted between October 2025 and February 2026 with 200 nurses working in the surgical clinics of a private hospital in Türkiye. Data were collected through digital forms by using the Socio-Demographic Data Form and the Attitude Scale Toward Non-Pharmacological Techniques in Postoperative Pain Management. Descriptive statistics, independent samples *t*-test, and one-way ANOVA were used for data analysis (*p* < 0.05). **Results:** Approximately 59% of the nurses were aged 20–29 years, and 65.5% were female. 68% of the nurses used both pharmacological and non-pharmacological techniques in postoperative pain management, and 80% of them believed that non-pharmacological methods were effective. The mean total score obtained from the scale was 48.42 ± 9.70, indicating that nurses had a positive attitude toward the use of non-pharmacological techniques. Mean scores related to non-pharmacological techniques differed significantly according to gender, marital status, educational level, professional experience, and department (*p* < 0.05). **Conclusions:** Surgical nurses demonstrated positive attitudes toward non-pharmacological postoperative pain management. Non-pharmacological techniques should be integrated into clinical practice by nurses in pain management.

## 1. Introduction

Postoperative pain, associated with tissue or organ damage occurring during the surgical procedure, is one of the most common symptoms experienced by patients, significantly affecting their quality of life [1]. The Taxonomy Committee of the International Association for the Study of Pain (IASP) defines pain as “an unpleasant sensory and emotional experience associated with actual or potential tissue damage” [2]. In the nursing literature and practice: “Pain is whatever the experiencing person says it is, existing whenever and wherever the person says it does” [3].

Each year, approximately 240 million patients undergo surgical procedures worldwide, and nearly 70% of them experience moderate to severe postoperative pain [1]. Inadequate postoperative pain management may lead to serious pulmonary and cardiac complications, including thrombosis, infection and organ failure, resulting in delayed discharge and consequently increased healthcare costs [4,5]. Conversely, when adequate postoperative pain management is achieved, the recovery process accelerates, potential complications decrease, and the risk of chronic pain decreases [6,7].

Surgical nurses are healthcare professionals who play a critical role in the pain management process due to their continuous exchange with the patients. They are primarily responsible for assessing patients’ pain, planning appropriate interventions, implementing these interventions, and ensuring the effective management of the entire process [8,9].

In the current general practice, postoperative pain management predominantly relies on the use of pharmacological agents. Studies have shown that nurses largely prefer pharmacological interventions in pain management [10]. However, the extensive use of pharmacological agents may cause several adverse effects, including nausea, vomiting, dizziness, sedation, and drug addiction [7,11]. As both techniques have their own benefits to achieve effective pain management, it is important to integrate non-pharmacological techniques alongside pharmacological treatments [8,12]. These methods offer several advantages, including the absence of side effects, ease of application, the opportunity for nurses to exercise their independent roles, and the enhancement of patients’ autonomy and comfort [13]. Surgical nurses can implement a wide range of non-pharmacological interventions as part of pain management. Commonly used approaches include massage, aromatherapy, music therapy, hot and cold applications, relaxation techniques, and patient education. In addition, providing emotional support and reassurance, therapeutic touch, and creating a comfortable environment are recognized as important nursing interventions [11]. Nevertheless, nurses’ knowledge, attitudes, opinions, skills, and experiences regarding these approaches significantly influence the use of non-pharmacological techniques in pain management [5]. Previous studies indicate that nurses often have insufficient knowledge and negative attitudes toward non-pharmacological pain management techniques, and many do not incorporate them into clinical practice [14]. Thus, assessing surgical nurses’ attitudes toward these methods and their actual patterns of use in clinical practice is crucial for improving the quality of nursing care and strengthening evidence-based pain management practices. Attitudes toward non-pharmacological pain management refer to nurses’ beliefs, perceptions, and willingness to use evidence-based non-pharmacological methods as part of routine pain care. These attitudes, being a significant determinant in clinical practice, show that nurses with positive attitudes are more likely to integrate nonpharmacological strategies into multifaceted pain management, educate patients about these approaches, and provide individualized pain care. Consequently, nurses’ attitudes play a crucial role in improving pain control, enhancing patient comfort and satisfaction, reducing pain-related complications, and delivering holistic nursing care. This study aimed to describe surgical nurses’ attitudes toward the use of non-pharmacological pain management techniques in surgery.

## 2. Research Questions

1. What are the attitudes of surgical nurses toward the use of non-pharmacological techniques in postoperative pain management?
2. How frequently do surgical nurses use non-pharmacological techniques in pain management?
3. Is there a relationship between surgical nurses’ attitudes and their use of non-pharmacological pain management techniques?

## 3. Materials and Methods

### 3.1. Study Design and Sample

This descriptive and cross-sectional study was conducted between October 2025 and February 2026 with nurses working in the surgical units of a private hospital in Türkiye. The sample size required to represent the population was calculated using a priori power analysis with G*Power 3.1 software. The sample size analysis was conducted using a significance level of 5% (α = 0.05), a statistical power of 85% (1 − β = 0.85), and a small, anticipated effect size (r = 0.15). A small effect size was selected as a conservative assumption to ensure adequate statistical power in the absence of previous studies reporting comparable effect sizes for the primary outcome. According to the power analysis, the minimum required sample size was determined to be 150 nurses. Additionally, the hospital where the study was conducted had 300 nurses working in its surgical units; thanks to the digital form being formatted in a way that did not allow for any questions to be skipped, the study was completed with 200 nurses and without any missing data.

**The inclusion criteria for the study:**

- Working as a surgical nurse,
- Having at least one year of active clinical experience in a surgical unit.

The exclusion criterion was withdrawing from the study at any stage of the research process.

### 3.2. Data Collection Tools

The data for the study were collected by using the Socio-Demographic Data Form and the Attitude Scale Toward Non-Pharmacological Techniques in Postoperative Pain Management.

#### 3.2.1. Socio-Demographic Data Form

This form was developed by the researchers in accordance with the literature [1,11] to determine the socio-demographic and professional characteristics of the participating nurses, such as age, sex, educational level, professional experience, unit of employment, etc.

#### 3.2.2. The Attitude Scale Toward Non-Pharmacological Techniques in Postoperative Pain Management

This scale is a valid and reliable instrument developed to assess the attitudes of nurses working in surgical and surgical intensive care units toward non-pharmacological postoperative pain management [15]. The scale consists of 13 items across three subdimensions: Approach (items 1–6), Continuation of Use (items 7–9), and Motivating Factors (items 10–13). It employs a 5-point Likert-type response format, with a total score ranging from 13 to 65. Higher scores indicate a more positive attitude, whereas lower scores indicate a more negative attitude. The scale’s Cronbach’s alpha coefficient is 0.88, indicating a high level of reliability [15]. In this study, the Cronbach’s alpha coefficient of the scale was calculated as 0.883. When the sub-dimensions were examined, the approach dimension was found to be 0.923, the continued use dimension 0.914, and the motivating factors 0.911.

### 3.3. Data Collection

The participants were visited and provided with information about the study in their relevant units, in order to not interrupt their workflow, and those who accepted were invited to fill the digital form via preferred communication platform. After the written consent, the data collection process started, and the researchers provided explanatory information only when necessary, without intervening or supporting independent responses. Completing the questionnaires took approximately 15–20 min. In accordance with ethical principles, personal data were carefully protected, and the questionnaires were coded using unique identification numbers rather than participants’ names. The code list was accessible only to the principal investigator.

### 3.4. Ethical Considerations

After ethical approval from the University’s Non-Interventional Ethics Committee (Date: 2 October 2025, No: 08/82), institutional permission was also obtained (Date: 25 September 2025). Informed consent was obtained from all participants. All stages of the research were conducted in accordance with the ethical principles of the World Medical Association’s Declaration of Helsinki.

### 3.5. Data Analysis

The research data were analysed using the Statistical Package for the Social Sciences (SPSS) version 27.0 (IBM Corp., Armonk, NY, USA) Categorical variables were presented as frequency and percentage (N, %), while continuous variables were expressed as mean ± standard deviation, median, and range (minimum–maximum). The Kolmogorov–Smirnov test was used to test the normality of scores obtained from a continuous variable using a statistical method. Comparisons between two groups for continuous variables were conducted using the independent samples *t*-test, whereas comparisons among more than two groups were performed using one-way ANOVA. In cases where a significant difference was found in the ANOVA analysis, the Tukey multiple comparison test was used to determine which groups were responsible for the difference. Statistical significance was evaluated at a 95% confidence interval, with *p* < 0.05 (two-sided) considered significant.

To examine the relationship between surgical nurses’ attitudes toward non-pharmacological pain management and their pain management techniques, multinomial logistic regression analysis was performed. Odds ratios (ORs) with 95% confidence intervals (95% CIs) were calculated, and statistical significance was set at *p* < 0.05. Multiple linear regression analysis was applied to determine the factors independently influencing attitude scores. In the analysis, the total attitude score was included as the dependent variable, and gender, marital status, education level, clinical experience, and professional experience were included as independent variables using the Enter method. Multicollinearity, tolerance, and VIF values were used for evaluation.

## 4. Results

According to the demographic characteristics of the participating nurses, 59% of them were aged between 20 and 29 years, 65.5% were female, and 58% were single. 83% of the nurses held a bachelor’s degree. Most of the nurses worked in the General Surgery (31%) and Orthopaedics (26%) departments. 80.5% of the nurses worked as staff nurses, and 58% worked in rotating shifts (Table 1).

An examination of nurses’ practices in pain management revealed that 82.5% had previously received training on the subject, with 60% of these trainings been provided as part of undergraduate education. In postoperative pain management, 68% of nurses use pharmacological and non-pharmacological techniques concurrently. The most frequently applied non-pharmacological intervention was hot–cold application (49%), followed by massage (27%). Eighty percent of nurses believe that non-pharmacological techniques are effective (Table 2).

The mean total score of the nurses on the scale was 48.42 ± 9.70. Examination of the subdimensions revealed mean scores of 22.45 ± 5.69 for the Approach, 10.81 ± 3.53 for the Continuation of Use, and 15.16 ± 4.00 for the Motivating Factors subdimension (Table 3).

The comparison of the scale scores by socio-demographic characteristics showed that the total mean score of the scale was significantly higher among female nurses (49.71 ± 9.32) compared to male nurses (45.97 ± 10.01) (t = 2.628; *p* = 0.009). There was also a significant difference in the Approach subdimension of the scale between the mean score of female nurses (23.18 ± 5.23) and that of male nurses (21.07 ± 6.28) (t = 2.517; *p* = 0.013). However, no significant differences were found between sexes in the Continuation of Use and Motivating Factors subdimensions (*p* > 0.05). Married nurses had a significantly higher mean Approach score (23.48 ± 5.30) than single nurses (21.71 ± 5.86) (t = 2.190; *p* = 0.030). In addition, in the Motivating Factors subdimension, married nurses had significantly higher scores (15.85 ± 3.95) than single nurses (14.66 ± 3.98) (t = 2.078; *p* = 0.039), while no significant differences were found in the Continuation of Use subdimension or in the total score according to marital status (*p* > 0.05). According to educational level, significant differences were identified among groups in the Continuation of Use (F = 3.753; *p* = 0.025) and Motivating Factors (F = 4.142; *p* = 0.017) subdimensions, with vocational health high school graduates and nurses holding master’s degrees demonstrating higher mean scores than those with bachelor’s degrees; however, no significant difference was observed in the total score (*p* = 0.142). In terms of professional experience, significant differences were found in the Approach subdimension (F = 4.516; *p* = 0.012) and total score (F = 3.375; *p* = 0.036), with the highest mean scores observed among nurses with 10–19 years of experience, whereas no significant differences were identified in the Continuation of Use and Motivating Factors subdimensions (*p* > 0.05).

Comparisons between clinical units showed that significant differences were identified in the Continuation of Use (F = 3.185; *p* = 0.015) and Motivating Factors (F = 3.289; *p* = 0.012) subdimensions. Nurses working in the cardiovascular surgery clinic had higher mean scores compared to those in other clinics, whereas nurses working in the urology clinic had lower mean scores. However, no significant difference was observed in terms of the total scale score (*p* = 0.172).

In conclusion, the mean scores related to non-pharmacological postoperative pain management were found to be influenced by sex, marital status, educational level, professional experience, and the clinical unit in which the nurses worked, whereas they were not affected by age, length of employment at the current institution, job position, or working schedule (*p* < 0.05) (Table 4).

Nurses who had received training had significantly higher mean scores in the Approach (22.87 ± 5.83) and Motivating Factors (15.42 ± 3.95) subdimensions, as well as higher total mean scores (49.12 ± 9.66), compared with nurses who had never received such training (*p* = 0.024, *p* = 0.047, *p* = 0.027, respectively). However, no significant difference was found in the Continuation of Use subdimension (*p* = 0.860).

Analysis based on the type of training revealed significant differences among groups in the Approach (F = 3.969; *p* = 0.009) and Motivating Factors (F = 4.212; *p* = 0.006) subdimensions, as well as in the total score (F = 4.069; *p* = 0.008). Nurses who had attended a congress had the highest total mean score (50.94 ± 10.47), while the group that had not received any training had the lowest total score (43.88 ± 8.90).

When asked about the most frequently used method in pain management, nurses who used both pharmacological and non-pharmacological techniques had higher total mean scores (50.19 ± 9.67) compared to other groups, whereas the group using only non-pharmacological techniques had a lower total mean score (40.83 ± 9.75).

Lastly, nurses who believed that non-pharmacological techniques are effective had a significantly higher total mean score (50.49 ± 9.19) compared to those who did not believe (40.28 ± 5.59) or were undecided (40.00 ± 7.93).

In conclusion, having received training in pain management, the type of training, the techniques used in pain management, belief in the effectiveness of non-pharmacological techniques, and the frequency of application significantly affected nurses’ mean scores for attitudes toward non-pharmacological pain management (*p* < 0.05) (Table 5).

According to multinomial logistic regression analysis, as attitude scores increase, the likelihood of using both pharmacological and non-pharmacological methods together significantly increases (OR = 1.047, *p* = 0.014). Conversely, the likelihood of preferring only non-pharmacological methods decreases significantly compared to the reference category (OR = 0.930, *p* = 0.039) (Table 6).

Multiple linear regression analysis was conducted to identify factors independently influencing the total attitude score. The analysis revealed that only gender was an independent predictor of the total attitude score (B = −3.656; β = −0.180; *p* = 0.011). Marital status (*p* = 0.092), education level (*p* = 0.136), department (*p* = 0.978), and professional experience (*p* = 0.714) were not independently associated with the total attitude score. No multicollinearity was observed (VIF = 1.017–1.162) (Table 7).

## 5. Discussion

Surgical nurses play a key role in the pain management process; therefore, nurses’ knowledge and attitudes have a valuable impact on the use of non-pharmacological techniques in pain management [2,11,12].

Among the surgical nurses participating in the study, 59% were aged between 20 and 29 years, and 65.5% were female. Similarly, in a study by Şişman et al., (2024) investigating the knowledge and behaviours of adult intensive care nurses regarding pain, 71.8% of the nurses were aged between 18 and 30 years, and 58.5% of them were female [12]. In a study conducted by Mohamed Bayoumi et al. (2021), the mean age of the nurses was 28.98 years, and more than one-third were females [16].

When examining nurses’ competencies in pain management, Mohamed Bayoumi et al. (2021) reported that a large proportion of nurses acquired sufficient knowledge about pain during their undergraduate nursing education [16]. Jira et al. (2020) found that 51.2% of nurses had adequate knowledge regarding non-pharmacological pain management [11]. Another study showed that 74 nurses (43.8%) received education on pain treatment during their formal training, but 100 nurses (56.2%) had not received any post-graduation training on the topic [17]. In this study, 82.5% of nurses had received pain management training, and the majority of this training (60.0%) was provided during the undergraduate studies. Notably, the rate of in-service training was relatively low (15.5%), indicating a gap in professional development for nurses after graduation. Evidence-based education on non-pharmacological pain management has been shown to enhance nurses’ knowledge and confidence and to support the clinical application of techniques aiming pain relieve [18]. These findings highlight the need to increase in-service training opportunities.

A study conducted with 443 surgical nurses working in various regions of Türkiye found that nearly all nurses applied non-pharmacological techniques for postoperative pain management [19]. Another study involving nurses working in intensive care units showed that 62.1% of nurses used both pharmacological and non-pharmacological techniques, while 33% applied only pharmacological techniques [20]. Çetinkaya et al. (2023), in a study investigating nurses’ knowledge regarding pain management and the use of non-pharmacological techniques, reported that 52.1% of nurses employed both pharmacological and non-pharmacological techniques in pain management [17]. In contrast, Türker and Şanlı (2024) found that nurses predominantly used pharmacological techniques and applied non-pharmacological techniques less frequently [21]. In this study, nurses used both pharmacological and non-pharmacological techniques in pain management at a rate of 68.0%. Additionally, 80.0% of nurses believed that non-pharmacological techniques were effective. These results suggest that the feasibility of implementing non-pharmacological pain management techniques may vary depending on the characteristics of the units where nurses work, their level of education, and cultural factors.

Commonly used non-pharmacological techniques in pain management include massage, positioning, hot–cold applications, relaxation exercises, and aromatherapy [12]. In this study, the most frequently applied technique was hot–cold application, while massage, music therapy, relaxation exercises, and distraction techniques were used less frequently. Similarly, Gümüş et al. (2020), in a study with a comparable sample, reported that hot–cold application was the most frequently used non-pharmacological technique for postoperative pain management, whereas reflexology was the least implemented [19]. Bilen and Balcı (2020) also observed that the most commonly used non-pharmacological techniques by nurses were massage and hot–cold application [22]. Mohamed Bayoumi et al. (2021) and Kidanemariam et al. (2020) found a different technique used frequently by nurses, which was cognitive-behavioural therapy [16,23]. Kiaa et al. (2021) reported that positioning was the most commonly used non-pharmacological intervention, whereas acupuncture and reflexology were applied less frequently [24]. The differences in applied techniques might stem from factors such as the cultural characteristics of the institutions where nurses work, their level of training regarding these techniques, and the workload in the clinical settings.

Evaluating nurses’ attitudes toward non-pharmacological practices in postoperative pain management is critically important for improving the quality of care. In a study conducted by Abimbola et al. (2021), it was reported that 65.7% of nurses had positive attitudes toward pain management practices, and a significant relationship was found between these attitudes and the use of non-pharmacological interventions [25]. Similarly, Tekletsadik et al. (2021), in their study investigating nurses’ knowledge and attitudes toward non-pharmacological pain management, found that 49.8% of nurses had a positive attitude and that the overall mean attitude score was 70% [18]. In this study, nurses’ attitudes toward non-pharmacological techniques in postoperative pain management were accurately measured by a reliable scale, and the result (48.42 ± 9.70) indicates that nurses demonstrated a positive attitude toward non-pharmacological interventions. This finding is consistent with the literature. The adoption and implementation of non-pharmacological techniques by surgical nurses not only enhance the effectiveness of pain control but also play a strategic role in managing the postoperative recovery process by helping to prevent surgical complications. According to this study’s results, surgical nurses tend to continue using non-pharmacological techniques in postoperative pain management and the motivating factors for these practices are high. Regarding attitudes, Tekletsadik et al. (2021) reported that 49.8% of nurses had a positive attitude toward non-pharmacological pain management, and similarly, Jira et al. (2020) found that 47% of nurses demonstrated a positive attitude [11,18]. Nurses’ tendency to continue using these methods can be explained by clinical experience, increased patient satisfaction, and access to low-cost interventions. However, previous studies have also indicated that time constraints, high workloads, staff shortages, and patients’ reluctance to use pharmacological techniques limit the application of these interventions [16,24,26,27]. From this perspective, the positive attitude observed in this study may be associated with the sample’s level of education, institutional support, or clinical experience. In conclusion, the positive attitudes and tendency of surgical nurses to continue using non-pharmacological techniques suggest that these practices can play a meaningful role in pain management within surgical clinics.

This study’s results demonstrated that nurses who had previously received training in pain management had significantly higher mean scores in the Approach, Motivating Factors subdimensions, and total score compared to those who had not received training, suggesting that education plays a key role in developing positive attitudes toward non-pharmacological techniques. According to the literature, nurses who attend courses on both pharmacological and non-pharmacological pain management achieve higher scores than those who do not participate in such courses [18]. Studies have also indicated that nurses trained in non-pharmacological pain management are approximately 7.5 times more likely to have adequate knowledge compared to those without such training [11]. Participation in educational courses provides nurses with opportunities to review various resources and literature and to access updated information on non-pharmacological pain management, which likely contributes to their higher attitude scores toward these practices.

### Limitations

The data in the study were obtained based on nurses’ self-reports, which may introduce some response bias. Additionally, as the sample size was limited, the findings of this study may not be generalizable to nurses in other specialties or to medical institutions with different pain management resources. Regional differences in clinical practice models, institutional policies, and organizational culture can influence nurses’ attitudes toward and use of non-pharmacological pain management techniques. The study was conducted at a single private hospital, which may limit the generalizability of the findings to other healthcare settings.

## 6. Conclusions

In this research, surgical nurses reflected a positive attitude toward non-pharmacological techniques in postoperative pain management.

Non-pharmacological pain management techniques are suitable, simple, and side-effect-free complementary approaches that can be used to alleviate pain in surgical clinics and can also be applied after patient discharge.

When applied effectively, these techniques can enhance the quality of care, patient comfort, reduce the side effects of sedation and analgesia, and minimize hospitalisation costs.

## Figures and Tables

**Table 1:** Socio-Demographic Data of the Nurses (N = 200).

	n	%
**Age (years)**		
20–29	118	59
30–39	53	26.5
40–49	29	14.5
**Sex**		
Female	131	65.5
Male	69	34.5
**Marital status**		
Married	84	42
Single	116	58
**Education status**		
Vocational Health High School	12	6
Undergraduate	166	83
Graduate	22	11
**Professional experience (years)**		
1–9	153	76.5
10–19	34	17
20–29	13	6.5
**Work duration in the present institution (years)**		
0–5	164	82
6–10	25	12.5
11–30	11	5.5
**Department**		
General Surgery	62	31
Orthopaedics	52	26
Urology	24	12
Cardiovascular Surgery	34	17
Ophthalmology	28	14
**Title**		
Staff Nurse	161	80.5
Head Nurse	39	19.5
**Working Schedule**		
Day Shift	84	42
Rotating Shifts	116	58

**Table 2:** Nurses’ General Practices on Pain (N = 200).

	n	%
**Have you ever received any training regarding pain management before?**		
Yes	165	82.5
No	35	17.5
**If Yes, what kind of education?**		
Undergraduate course content	120	60
In-service training	31	15.5
Congress/Seminar	17	8.5
Others	32	16
**What is the technique you most frequently use in postoperative pain management?**		
Pharmacological technique	46	23
Non-pharmacological technique	18	9
Both	136	68
**What is the non-pharmacological technique you most frequently use in postoperative pain management?**		
Hot/cold application	98	49
Massage	54	27
Music therapy	26	13
Relaxation exercises	19	9.5
Distraction	3	1.5
**Do you believe that non-pharmacological techniques are effective in postoperative pain management?**		
Yes	160	80
No	18	9
Undecided	22	11
**What is the frequency of the application of these techniques?**		
Always	35	17.5
Frequently	67	33.5
Sometimes	72	36
Rarely	24	12
Never	2	1

**Table 3:** Mean Scores of the Subdimensions and Total Scores of the Attitude Scale Toward Non-Pharmacological Practices in Postoperative Pain Management.

	Min-Max	Mean ± SD
Approach	6–30	22.45 ± 5.69
Continuation of Use	3–15	10.81 ± 3.53
Motivating Factors	4–20	15.16 ± 4.00
Total	13–65	48.42 ± 9.70

SD: Standard Deviation.

**Table 4:** Relationship Between Socio-Demographic Characteristics and Attitudes Scale Toward Non-Pharmacological Techniques in Postoperative Pain Management Mean Scores (N = 200).

Variables	Category	Approach	Continuation of Use	Motivating Factors	Total
**Age (years)**	20–29	22.14 ± 6.04	10.70 ± 3.49	15.0 ± 4.05	47.84 ± 9.70
	30–39	23.32 ± 5.53	10.62 ± 3.87	15.94 ± 3.84	49.89 ± 9.83
	40–49	22.14 ± 4.30	11.59 ± 2.98	14.38 ± 3.98	48.10 ± 9.53
	**Test value ^b^**	0.653 ^b^	1.053 ^b^	1.558 ^b^	0.966 ^b^
	***p* value**	0.912	0.401	0.277	0.521
Sex	Female	23.18 ± 5.23	11.15 ± 3.46	15.38 ± 3.90	49.71 ± 9.32
	Male	21.07 ± 6.28	10.16 ± 3.59	14.74 ± 4.18	45.97 ± 10.01
	**Test value ^a^**	2.517 ^a^	1.904 ^a^	1.080 ^a^	**2.628 ^a^**
	***p* value**	0.013 **	0.058	0.282	0.009 **
Marital status	Married	23.48 ± 5.30	10.73 ± 3.84	15.85 ± 3.95	50.05 ± 9.85
	Single	21.71 ± 5.86	10.87 ± 3.29	14.66 ± 3.98	47.24 ± 9.46
	**Test value ^a^**	2.190 ^a^	0.285 ^a^	2.078 ^a^	2.034 ^a^
	***p* value**	0.030 **	0.776	0.039	0.403
Education status	Vocational Health High School	21.42 ± 4.96	12.75 ± 1.54	17.50 ± 2.27	51.67 ± 6.82
	Undergraduate	22.49 ± 5.68	10.51 ± 3.61	14.81 ± 4.09	47.81 ± 9.87
	Graduate	22.73 ± 6.29	12.00 ± 3.17	16.55 ± 3.32	51.27 ± 9.21
	**Test value ^b^**	0.226 ^b^	3.753 ^b^	4.142 ^b^	1.972 ^b^
	***p* value**	0.798	0.025 **	0.017 **	0.142
Professional experience	1–9 (1)	22.08 ± 5.80	10.73 ± 3.46	15.15 ± 3.92	47.95 ± 9.44
	10–19 (2)	24.91 ± 5.20	11.12 ± 3.99	15.88 ± 4.14	51.91 ± 10.61
	20–29 (3)	20.38 ± 3.47	11.00 ± 3.18	13.38 ± 4.29	44.77 ± 8.47
	**Test value ^b^**	4.516 ^b^	0.190 ^b^	1.849 ^b^	3.375 ^b^
	***p* value**	0.012 **	0.827	0.160	0.036 **
	***Tukey Test: 1,3 < 2***				
Work duration in the present institution	0–5	22.20 ± 5.75	10.74 ± 3.51	15.12 ± 4.01	48.05 ± 9.55
	6–10	23.76 ± 5.33	10.36 ± 3.92	15.12 ± 4.30	49.24 ± 10.55
	11–30	23.27 ± 5.49	12.82 ± 2.27	15.91 ± 3.36	52.00 ± 10.06
	**Test value ^b^**	0.941 ^b^	2.032 ^b^	0.202 ^b^	0.953 ^b^
	***p* value**	0.392	0.134	0.817	0.387
Department	General Surgery (1)	22.48 ± 5.93	10.85 ± 3.67	15.32 ± 3.78	48.66 ± 8.77
	Orthopaedics (2)	22.87 ± 5.03	10.29 ± 3.81	14.79 ± 4.62	48.94 ± 10.69
	Urology (3)	21.54 ± 5.29	9.25 ± 3.28	13.25 ± 3.42	44.04 ± 9.90
	Cardiovascular surgery (4)	21.12 ± 7.28	12.29 ± 2.94	16.91 ± 3.34	50.32 ± 10.05
	Ophthalmology (5)	22.14 ± 3.98	11.21 ± 2.91	15.00 ± 3.77	48.36 ± 8.66
	**Test value ^b^**	1.146 ^b^	3.185 ^b^	3.289 ^b^	1.615 ^b^
	***p* value**	0.217	0.015 **	0.012 **	0.172
	***Tukey Test: 3 < 4***				
Title	Staff Nurse	22.61 ± 5.62	10.70 ± 3.42	14.99 ± 3.99	48.30 ± 9.80
	Head Nurse	21.77 ± 6.0	11.28 ± 3.96	15.87 ± 4.0	48.92 ± 9.41
	**Test value ^a^**	0.832 ^a^	0.930 ^a^	1.240 ^a^	0.360 ^a^
	***p* value**	0.407	0.353	0.217	0.719
Working schedule	Day Shift	21.86 ± 4.91	11.14 ± 3.21	15.18 ± 3.72	48.18 ± 9.06
	Rotating Shifts	22.88 ± 6.17	10.57 ± 3.73	15.15 ± 4.20	48.59 ± 10.18
	**Test value ^a^**	1.255 ^a^	1.135 ^a^	0.056 ^a^	0.299 ^a^
	***p* value**	0.211	0.258	0.956	0.765

** *p* < 0.05; ^a^ (*t*), Independent sample *t*-test; ^b^ (*F*), One way ANOVA test; SD; standard deviation.

**Table 5:** Relationship Between Pain-Related Questions and Attitudes Toward Non-Pharmacological Techniques in Postoperative Pain Management Mean Scores (N = 200).

Variables	Category	Approach	Continuation of Use	Motivating Factors	Total
Have you ever received any training regarding pain management before?	Yes	22.87 ± 5.83	10.83 ± 3.62	15.42 ± 3.95	49.12 ± 9.66
	No	20.49 ± 4.55	10.71 ± 3.10	13.94 ± 4.05	45.14 ± 9.34
	**Test value ^a^**	0.653 ^a^	1.053 ^a^	1.558 ^a^	0.966 ^a^
	***p* value**	0.024 **	0.860	0.047 **	0.027 **
If Yes, what kind of education?	Undergraduate course content (1)	23.53 ± 5.77	10.74 ± 3.78	15.50 ± 3.84	49.77 ± 9.42
	In-service training (2)	21.26 ± 4.52	10.71 ± 3.28	14.55 ± 4.31	46.52 ± 9.75
	Congresses/Seminar (3)	21.29 ± 7.47	12.47 ± 2.55	17.18 ± 3.52	50.94 ± 10.47
	Others (4)	20.19 ± 4.40	10.28 ± 3.05	13.41 ± 3.92	43.88 ± 8.90
	**Test value ^b^**	3.969 ^b^	1.528 ^b^	4.212 ^b^	4.069 ^b^
	***p* value**	0.009 **	0.208	0.006 **	0.008 **
	***Tukey Test: 1,3 > 4***				
The technique you most frequently use in pain management?	Pharmacological methods (1)	23.48 ± 5.30	11.20 ± 2.76	15.02 ± 3.49	46.15 ± 7.89
	Non-pharmacological methods (2)	21.71 ± 5.86	9.61 ± 2.50	13.00 ± 3.41	40.83 ± 9.75
	Both (3)	23.86 ± 5.29	10.84 ± 3.84	15.49 ± 4.16	50.19 ± 9.67
	**Test value ^b^**	15.635 ^b^	1.321 ^b^	3.188 ^b^	9.819 ^b^
	***p* value**	0.000 **	0.269	0.043 **	0.000 **
	***Tukey Test: 1,2 < 3***				
Which non-pharmacological technique you most frequently use in pain management?	Hot/cold application (1)	23.32 ± 5.25	10.82 ± 3.52	15.46 ± 4.08	49.59 ± 9.80
	Massage (2)	21.98 ± 5.66	10.76 ± 3.41	15.31 ± 3.54	48.06 ± 8.92
	Music therapy (3)	19.85 ± 6.56	11.08 ± 3.24	14.31 ± 3.60	45.23 ± 9.86
	Relaxation exercises (4)	24.00 ± 4.70	10.21 ± 4.46	13.89 ± 5.20	48.11 ± 10.69
	Distraction (5)	15.33 ± 8.08	13.00 ± 2.64	18.00 ± 1.73	46.33 ± 12.05
	**Test value ^b^**	3.379 ^b^	0.460 ^b^	1.313 ^b^	1.120 ^b^
	***p* value**	0.006 **	0.765	0.267	0.348
	***Tukey Test: 1 > 3***				
Do you believe that non-pharmacological techniques are effective in pain management?	Yes (1)	23.49 ± 5.61	11.20 ± 3.71	15.81 ± 3.98	50.49 ± 9.19
	No (2)	18.67 ± 3.30	8.94 ± 1.62	12.67 ± 2.54	40.28 ± 5.59
	Undecided (3)	18.00 ± 4.25	9.50 ± 2.30	12.50 ± 3.18	40.00 ± 7.93
	**Test value ^b^**	4.516 ^b^	0.190 ^b^	1.849 ^b^	3.375 ^b^
	***p* value**	0.000 **	0.006	0.000	0.000 **
	***Tukey Test: 1 > 2,3***				
How often do you apply these techniques?	Always (1)	23.14 ± 7.10	9.71 ± 4.16	15.40 ± 4.94	48.26 ± 10.47
	Frequently (2)	24.10 ± 5.27	11.36 ± 3.96	15.88 ± 4.28	51.34 ± 9.85
	Sometimes (3)	21.99 ± 5.12	11.18 ± 3.02	15.21 ± 3.32	48.38 ± 8.50
	Rarely (4)	18.75 ± 4.16	9.79 ± 2.10	13.00 ± 2.76	41.54 ± 8.09
	Never (5)	16.00 ± 2.82	10.50 ± 2.12	11.00 ± 1.41	37.50 ± 2.12
	**Test value ^b^**	5.256 ^b^	1.986 ^b^	2.980 ^b^	5.652 ^b^
	***p* value**	0.000 **	0.098	0.020 **	0.000 **
	***Tukey Test: 1,2 > 4***				

** *p* < 0.05; ^a^: Independent sample *t*-test; ^b^: One way ANOVA test; SD: standard deviation.

**Table 6:** Multiple Logistic Regression Analysis of the Relationship Between Total Attitude Score and Pain Management.

Variable	Comparison	B	SE	OR (95% CI)	*p*
Total attitude score	Non-pharmacological vs. Pharmacological	−0.072	0.035	0.930 (0.869–0.996)	0.039
Total attitude score	Both vs. Pharmacological	0.046	0.019	1.047 (1.009–1.086)	0.014

B = regression coefficient; SE = standard error; OR = odds ratio; CI = confidence interval.

**Table 7:** Multivariate linear regression analysis of factors predicting caring behaviours.

Predictors	R	R^2^	Adjusted R^2^	B	SE	β	95% CI	F	*p*
**Multivariate model**	0.26	0.068	0.044						
Constant				59.192	3.652	–	51.990 to 66.395	–	0.001
Sex				−3.656	1.424	−0.180	−6.464 to −0.848	1.017	0.011
Marital status				−2.444	1.442	−0.125	−5.287 to 0.400	1.124	0.092
Education				−2.736	1.828	−0.106	−6.342 to 0.871	1.047	0.136
Department				−0.041	1.479	−0.002	−2.958 to 2.875	1.039	0.978
Professional experience				0.044	0.121	0.027	−0.194 to 0.283	1.162	0.714

B: Unstandardized regression coefficient; SE: Standard error; β: Standardized regression coefficient; R^2^: Explained variance; Adjusted R^2^: Adjusted explained variance; CI: Confidence interval.

## Data Availability

The datasets generated and/or analysed during the current study are not publicly available due to a language barrier, as the study is conducted in Turkish, and due to privacy reasons, but are available from the corresponding author on reasonable request.

## Public Involvement Statement

The participants were visited and provided with information about the study in their relevant units, in order to not interrupt their workflow, and those who accepted were invited to fill the digital form via preferred communication platform.

## Guidelines and Standards Statement

This manuscript was drafted against the STROBE reporting guidelines for cross-sectional research.
